# Digestive Enzyme Activity and Temperature: Evolutionary Constraint or Physiological Flexibility?

**DOI:** 10.3390/ani16010100

**Published:** 2025-12-29

**Authors:** Konstantinos Sagonas, Foteini Paraskevopoulou, Panayiota Kotsakiozi, Ilias Sozopoulos, Panayiotis Pafilis, Efstratios D. Valakos

**Affiliations:** 1 Department of Zoology, School of Biology, Aristotle University of Thessaloniki, 54124 Thessaloniki, Greece; fparas@bio.auth.gr; 2 Section of Animal and Human Physiology, Department of Biology, National and Kapodistrian University of Athens, 15784 Athens, Greece; pkotsakiozi@biol.uoa.gr (P.K.); isozop@biol.uoa.gr (I.S.); evalakos@biol.uoa.gr (E.D.V.); 3 Zoological Museum, National and Kapodistrian University of Athens, 15784 Athens, Greece; ppafil@biol.uoa.gr; 4 Section of Zoology and Marine Biology, Department of Biology, National and Kapodistrian University of Athens, 15784 Athens, Greece

**Keywords:** digestion, Greece, insularity, lizards, Mediterranean, *Podarcis*, proteases, lipases, maltases, temperature

## Abstract

Temperature strongly influences physiological processes in ectotherms, including digestion, yet its effects on digestive enzyme activity remain poorly understood. We examined the temperature dependence of digestive performance in eight Mediterranean wall lizard species (*Podarcis* spp.) from mainland and island populations. Under controlled laboratory conditions, we measured the activity of three key enzymes, protease, lipase, and maltase, across a temperature gradient (20–55 °C), alongside gastrointestinal (GI) morphology. Enzyme activity generally increased with temperature up to 50 °C and declined thereafter, reflecting typical thermal kinetics. Lipase activity was consistently higher in island species, while protease and maltase showed no significant geographic or phylogenetic trends. Island lizards also exhibited longer and heavier GI tracts relative to body size (SVL), suggesting enhanced nutrient absorption capacity. Phylogenetic signal analyses (Pagel’s λ and Abouheif’s *C*_mean_) revealed no significant evolutionary constraints on digestive traits, indicating that observed differences reflect ecological adaptation rather than ancestry. Overall, island species appear to have evolved digestive traits that improve energy extraction under resource-limited conditions, but may be more sensitive to extreme heat. These findings highlight contrasting adaptive strategies between island and mainland reptiles and underscore the importance of digestive physiology in predicting the response of species to warming climates.

## Simple Summary

Lizards rely on external temperatures to regulate their body functions, including digestion. This study explored how rising temperatures affect the activity of digestive enzymes, (protease, lipase, and maltase) in eight Mediterranean wall lizard species living on the Greek mainland and islands. Our findings showed that enzyme activity generally increased with temperature up to about 50 °C and then declined. Island species showed higher lipase activity than mainland species in addition to longer intestines, suggesting better fat digestion, an advantage in environments of food scarcity or unpredictability. However, island species also showed sharper drops in enzyme activities at very high temperatures, indicating lower heat tolerance. These results suggest that island and mainland lizard species have developed different digestive strategies to cope with their environments. Understanding these physiological differences helps predict how reptiles might respond to future variations in climate in terms of food intake and energy assimilation.

## 1. Introduction

Global environmental changes, including shifts in climate, habitat availability, and resource distribution, pose growing challenges to animal survival and adaptation [1]. Predicting species’ resilience under these conditions requires a deep understanding of the physiological mechanisms that determine their capacity to cope with environmental stressors. While much research has focused on thermotolerance and behavioral thermoregulation [2,3], less attention has been given to how rising temperatures affect digestive physiology, a key determinant of energy acquisition, metabolism, and overall fitness [4].

For ectothermic animals such as lizards, body temperature, and therefore digestive function, is largely dictated by external thermal conditions [4,5]. Digestive efficiency, defined as the ability to extract and assimilate nutrients from food, is central to energy balance and influences maintenance, growth, and reproduction [6]. As global temperatures are projected to rise between 1.4 °C and 5.8 °C by 2100 [7], lizards must either shift their distribution to thermally more favorable habitats or physiologically adjust to maintain digestive performance and energy balance [8,9]. Failure to do so may reduce digestive efficiency, forcing increased foraging effort, which could negatively affect survival and reproductive success [10].

Temperature influences digestive efficiency via multiple physiological mechanisms, including gastrointestinal (GI) motility [11], gut transit time [12], and enzymatic activity [13]. Digestive enzymes break down complex food molecules into absorbable units [13] and their activity generally follows temperature-dependent kinetics [4,14,15]. While enzymatic activity tends to increase with temperature up to an optimum, the extent to which different lizard species can maintain optimal digestive enzyme function under warming conditions remains unclear.

Proteases, lipases and carbohydrases are key digestive enzymes responsible for processing fats, sugars, and proteins, respectively [13]. Lipases catalyze the hydrolysis of fatty acid esters, essential for energy regulation in lizards [16]. Carbohydrases, such as alpha glucosidase (maltase), break down sugars into absorbable monosaccharides [17], while proteases hydrolyze dietary proteins into peptides and free amino acids and are particularly important in carnivorous species where they directly influence growth and tissue regeneration [4,18,19]. Despite their importance, most studies on the thermal sensitivity of digestive enzymes have focused on fish [20,21,22] and birds [23,24,25], leaving reptiles largely understudied.

In this study, under controlled laboratory conditions, we tested the response of digestive enzymes to in vitro incubation temperature increases in eight species of Mediterranean lacertid lizards that inhabit different environments, on the mainland and the islands. We focused on two key components of digestive performance: gastrointestinal (GI) tract morphology and the activity of proteases, lipase, and maltase. Our primary goals were to identify interspecific differences in enzyme thermal sensitivity, compare mainland and island populations, and assess whether observed variations in digestive performance [4] is driven by environmental adaptation or constrained by phylogeny. Building on the work of Pafilis et al. [4], who found that gut passage time and apparent digestive efficiency for proteins, sugars and lipids increased with temperature, especially in insular species, we hypothesized that island-dwelling lizards would exhibit enhanced enzyme activity and greater intestinal surface area (or length), reflecting adaptations for more efficient nutrient extraction in warmer and potentially more resource-limited environments.

## 2. Materials and Methods

### 2.1. Study Species and Area

**Figure 1:**
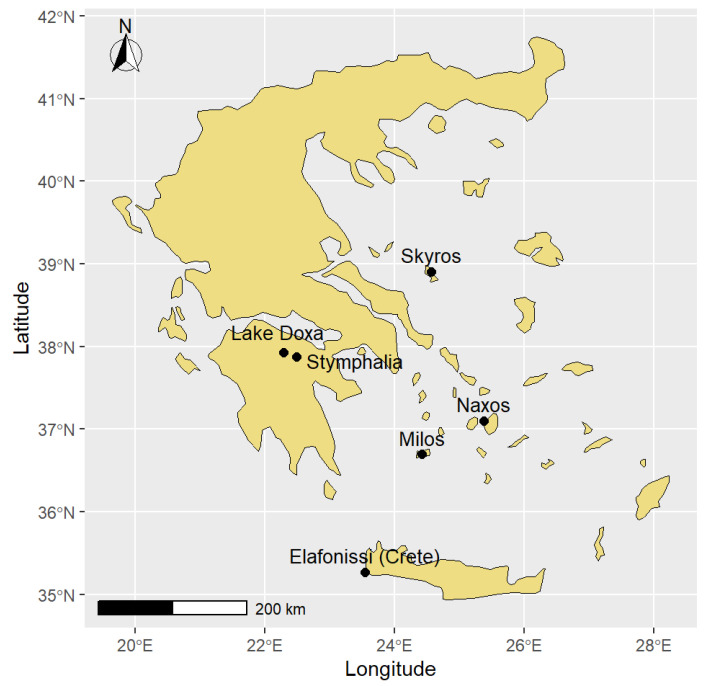
Map of Greece in the East Mediterranean Sea. Dots denote the six sampling locations. Naxos: *Podarcis erhardii*; Milos: *Podarcis milensis*; Crete: *Podarcis cretensis*; Skyros: *Podarcis gaigeae*; Lake Doxa: *Podarcis tauricus*, *Podarcis peloponessiacus*; *Podarcis muralis*; Stymphalia: *Podarcis thais*.

The study was conducted using eight species of wall lizards (genus *Podarcis*) (N = 152) inhabiting diverse Mediterranean habitats across mainland and insular Greece (Figure 1). All species are small-bodied, generalist arthropod predators with similar thermal preferences [26,27,28,29], and have known phylogenetic relationships [30,31,32]. While their diet primarily comprises terrestrial invertebrates, particularly insects, insular populations display additional dietary behaviors such as frugivory (fruit consumption), ovophagy (egg consumption), and herbivory [33].

**Figure 2:**
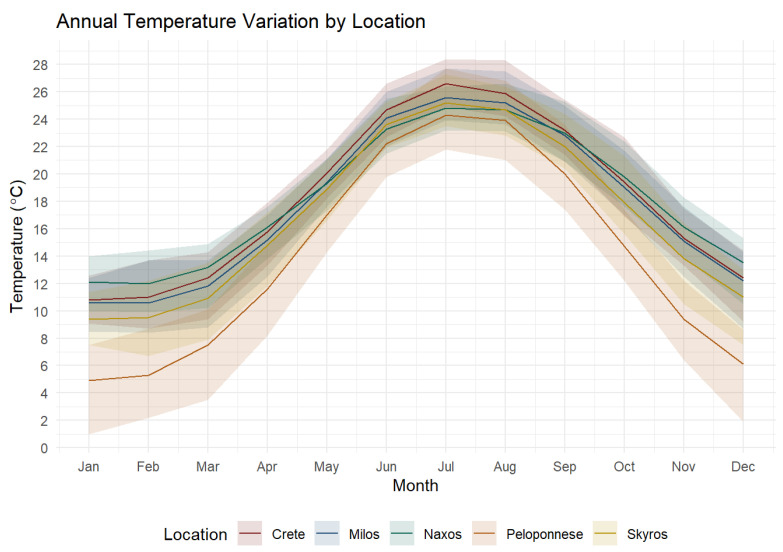
Monthly means of air temperature for the last 30 years, in six sampling locations. Crete (*P. cretensis*), Milos (*P. milensis*) Naxos (*P. erhardii*), Peloponnese (Lake Doxa for *P. peloponnesiacus* and Stymphalia for *P. thais*) and Skyros (*P. gaigeae*).

The three mainland species included the widely distributed common wall lizard (*P. muralis*; N = 18), the endemic Peloponnese wall lizard (*P. peloponnesiacus*; N = 22), and the Balkan wall lizard (*P. tauricus*; N = 18). These were collected from a narrow strip of land (100 m in length, 20 m in width) near Lake Doxa on the Feneos plateau (FE; 37.93112° N, 22.28341° E) (Figure 1). The area is surrounded by high mountains covered with forests of fir, black pine (*Pinus nigra*), oak (*Quercus coccifera*), and plane trees (*Platanus occidentalis*), and has a temperate climate with snowy and rainy winters and warm summers (Figure 2). The eastern Peloponnese wall lizard (*P. thais*; N = 18), endemic to the eastern Peloponnese, was sampled from the western banks of Lake Stymphalia (LS; 37.86836° N, 22.46054° E; Figure 1). The habitat at Stymphalia consists of a rocky slope 20 m from the lake, featuring many crevices and small overhangs with low plants (*Thymus vulgaris*, *Erica* sp., *Phlomis fruticosa*), while the base and top of the slope are covered by *Quercus coccifera*, *Arbutus unedo*, and *Spartium junceum*.

Among the island species (see Figure 1 for all island sampling locations), Erhard’s wall lizards (*P. erhardii*; N = 19), widely distributed across the southern Balkans and the Aegean islands, were sampled from Naxos Island (NX; 37.08294° N, 25.57855° E). The Skyros wall lizard (*P. gaigeae*; N = 21), endemic to the Skyros Archipelago and Piperi Island, was sampled from Skyros Island (SK; 38.82349° N, 24.56740° E). The Milos wall lizard (*P. milensis*; N = 18), endemic to the Milos Archipelago, was sampled from Milos Island (ML; 36.68658° N, 24.45722° E), while the Cretan wall lizard (*P. cretensis*; N = 18) was captured from Elafonissi at Crete (CR; 35.27098° N, 23.54615° E). The predominant vegetation of all island sites is the typical Mediterranean maquis and phrygana, primarily *Sarcopoterium spinosum* and *Thymus capitatus*, with a rocky substrate. The climate is milder and warmer than in continental areas [34], thanks to the buffering effect of the surrounding sea [35]. Monthly means of temperature for the last 30 years were obtained from the National Meteorological Service of Greece (http://climatlas.hnms.gr/sdi/, accessed on 29 October 2025) (Figure 2).

Lizards were collected by noosing during the nonreproductive period in September 2014 and in accordance with Greek National Law (Presidential Decree 67/81). To avoid possible sex and age effect, we used exclusively adult males. For each lizard collected, we recorded SVL (in mm) with a digital caliper (Silverline 380244, Silverline Tools, Walsall, UK, accurate to 0.01 mm) and body mass using a digital scale (i500 Backlit Display, My Weight, Digital Scales Company, Gloucestershire, UK, accurate to 0.1 g). One hundred and fifty-two lizards were captured in the field and transported to the animal facilities of the National and Kapodistrian University of Athens and housed individually in plastic terraria (20 cm × 25 cm × 15 cm) containing a sandy substrate and artificial shelters. The room temperature was kept at 25 °C and a controlled photoperiod (12 light: 12 dark) was provided by fluorescent bulbs, while additional incandescent lamps (60 W) and UVB lamps upon each terrarium allowed animals to thermoregulate behaviorally for 12 h/d. All lizards were fed mealworms (*Tenebrio molitor*) coated with mineral powder (TerraVit Powder, JBL GmbH & Co. KG, Neuhofen, Germany) every other day, and had access to water ad libitum.

### 2.2. Intestinal Tissue Preparation

One hundred and fifty-two lizards were captured and maintained under laboratory conditions, of which 144 individuals were processed for biochemical analyses. Prior to the experiment, lizards were fasted for 48 h to complete gut clearance [4] and to prevent residual food from interfering with enzyme assays. Following the fasting period, lizards were killed by rapid decapitation following deep anesthesia with isoflurane. After death, the gastrointestinal tract was immediately dissected, and any remaining food residues and fecal material were carefully removed from the intestinal lumen before tissue collection. In rare cases (n = 8) where undigested food remained within the lumen, samples were excluded from further analyses. This ensured that the subsequent estimate of enzymatic activities was based solely on the intestinal tissue itself, rather than any residual waste or food particles. Protease, lipase and maltase activities were measured in all processed individuals. Dissections were undertaken over ice to avoid enzyme and tissue degradation. The GI tract, spanning from the stomach to the cloaca, was cut lengthwise and rinsed with Ringer’s solution (0.1 M NaCl, 1.8 mM KCl, 2.0 mM CaCl_2_, 1.0 mM MgCl_2_, 5.0 mM HEPES-NaOH, pH 7.6). The length of the GI tract was then measured using digital calipers (to 0.01 mm) and weighed using a precision scale (wet mass, to 0.001 g). The tract was divided into three sections, based on standard anatomical landmarks: Section 1 (foregut; stomach), Section 2 (midgut; duodenum), and Section 3 (hindgut; the rest of the intestine extending to the cloaca). The pancreas was carefully excluded from all samples. Each section was weighed and measured again before being frozen in liquid nitrogen and stored at −80 °C for subsequent enzyme activity analysis. Intestine tissue samples were thawed at 21 to 23 °C and homogenized with 0.1 M phosphoric buffer pH 7.0. Tissue blanks were used as controls to distinguish enzymatic activity from baseline substrate concentrations. Standardized intestinal enzymatic activity was calculated on the basis of absorbance and expressed as μmoles × gr^−1^ × min^−1^ for each section.

### 2.3. Assays of Digestive Enzyme Activities

Protease, lipase, and maltase activities were assessed at 20, 25, 30, 33, 35, and 40 °C, temperatures within the thermal tolerance range of *Podarcis* lizards (e.g., [36,37]) and commonly recorded in the field during summer, as well as at 45, 50, and 55 °C [38,39], representing the upper extremes they might encounter. Protease activity was estimated using the Lowry method [40] with denatured casein (4% *w*/*v*) as the substrate, a well-established approach. This method quantifies total protein, including small peptides generated during digestion. Following careful tissue preparation and the removal of digesta to minimize confounding factors, this method can also serve as an indirect measure of protease activity through protein hydrolysis. Homogenized tissue was incubated for 30 min at the desired temperature (20–55 °C), and the reaction was terminated by adding 10% (*v*/*v*) trichloroacetic acid (TCA). The sample was centrifuged at 6000 rpm for 10 min; the pellet was discarded, and the supernatant was used to determine hydrolyzed protein. Folin phenol reagent was added to the sample, which was then incubated for another 30 min. Finally, the concentration of the reduced Folin reagent was measured at 750 nm, using a spectrophotometer (Barnstead/Turner SP-830 Plus, Turner, Madison, WI, USA).

To estimate total lipase activity, we used the oxidation of glycerol to dihydroxyacetone phosphate by glycerol kinase, a method modified from Kessler and Lederer [41]. While the original method was optimized to measure lipoprotein lipase activity in adipose tissue, skeletal muscle, and plasma, its principle of quantifying glycerol release from triglyceride hydrolysis is broadly applicable for assessing lipase activity. Here, using olive oil as a substrate to reflect the hydrolytic activity of lipases, the method was modified to suit intestinal tissue homogenates. Because the gastrointestinal tract was rinsed and the pancreas excluded, our measurements primarily reflect tissue-associated lipase activity in the intestinal mucosa (e.g., brush-border and intracellular lipases), rather than pancreatic secretions in the lumen. Although the assay does not distinguish among specific lipase types, the careful preparation of intestinal homogenates minimized contributions from luminal contents, and provides a consistent comparative index of triglyceride hydrolysis across populations. This approach thus offers valuable insights into overall enzymatic potential in the intestinal tissues of the studied lizards. The reaction was initiated by incubating the homogenized tissue at the desired temperature for 60 min with 0.5 mL of olive oil as the substrate. To terminate the reaction, samples were incubated at 100 °C for 10 min. The samples were then centrifuged at 3000 rpm for 5 min, and 0.5 mL of the supernatant was mixed with 2 mL of glycine phosphate buffer and 0.4 mL of NAD. Lipase activity was quantified based on the amount of released glycerol, determined by measuring the absorbance of NADH at 340 nm every 10 min until stabilization.

Maltase activity was measured using the glucose oxidase method (Glucinet kit; Glucofix, Menarini Diagnostics, Winnersh, UK). Homogenized tissue was incubated with 4% (*w*/*v*) maltose at a desired temperature (20–55 °C) for 30 min. The reaction was stopped by adding 10% (*v*/*v*) trichloroacetic acid (TCA), and the samples were centrifuged at 3000 rpm for 5 min. The resulting pellet was discarded. A universal pH indicator [Merck Universal Indicator (pH 4–10), Merck KGaA, Darmstadt, Germany] was then added, followed by a second centrifugation at 30,000 rpm for 5 min. Absorbance was measured at 510 nm using a spectrophotometer after 10 min of incubation at 37 °C. Additionally, we measured sucrose activity in each section of the gastrointestinal tract, but it was too low to be quantified.

### 2.4. Statistical Analysis

Data were analyzed in R version 4.3 [42]. Non-parametric tests were performed when parametric assumptions were not satisfied, and a significance level of α 0.05 was adopted, unless stated otherwise. A one-way analysis of variance (ANOVA) was used to compare body length, body mass, GI length and GI mass among populations. When necessary, an ANCOVA was performed, considering the SVL of each lizard as a covariate. Likewise, an ANOVA was performed to test differences in proteases, lipases and maltase activities between species across temperature treatments. Post hoc multiple comparisons were performed using the Tukey HSD method.

### 2.5. Phylogenetic Signal

The tendency for closely related species to share similarities in ecology, morphology, and physiology more than distantly related ones is known as phylogenetic signal [43]. This phylogenetic relatedness violates the assumptions of normal statistical methods, leading to the non-independence of trait values among species. To account for phylogenetic signal in our dataset, we used the Pagel’s λ [44] and Abouheif’s *C*_mean_ [45] methods, both of which are powerful and reliable for measuring and testing a phylogenetic signal [46] and have been widely applied to ecological traits [47,48,49]. Since phylogenetic signal estimates depend on the underlying phylogenetic tree, we constructed a phylogeny of the eight *Podarcis* species using mitochondrial and nuclear gene sequences available in the literature [30,31,32,50]. The robustness of the results was assessed using 1000 bootstrap replicates.

## 3. Results

### 3.1. Body Size and Gut Length

**Figure 3:**
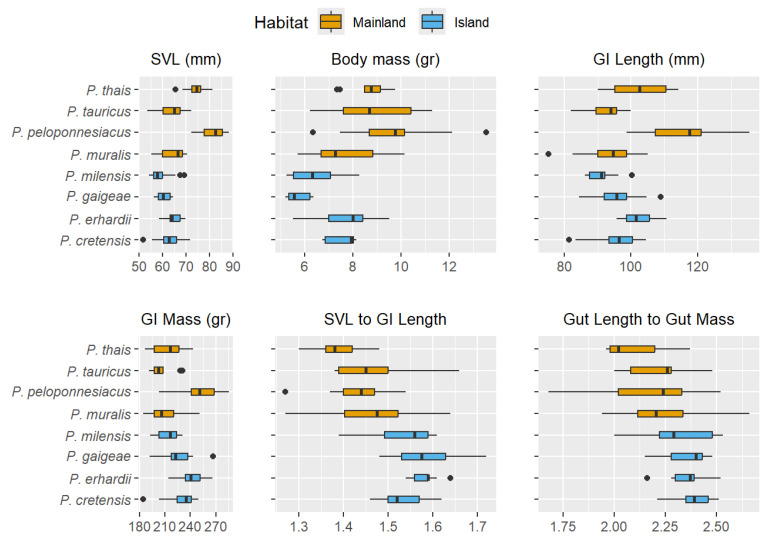
Box plot for all morphological traits examined. Blue bars correspond to mainland distributed species, while yellow bars represent insular *Podarcis* species and populations. SVL refers to snout-vent-length. Dots indicate outliers.

The comparison of body size (SVL; F_7,136_ = 41.18, *p* < 0.001), body mass (F_7,136_ = 17.68, *p* < 0.001), gut length (GILength; F_7,136_ = 22.46, *p* < 0.001) and gut mass (GIMass; F_7,136_ = 13.44, *p* < 0.001) revealed significant differences among the eight *Podarcis* species (Figure 3). Mainland species (*P. tauricus*, *P. peloponnesiacus*, *P. muralis*, and *P. thais*) exhibited significantly longer and heavier body and gut compared to island species. Since body length correlates positively with GI tract length (r^2^ = 0.76, t = 20.68, *p* < 0.001), and GI length with GI mass (r^2^ = 0.42, t = 10.22, *p* < 0.001), we calculated the ratios of these traits and repeated the analysis. Interestingly, although mainland lizards had overall larger GI tract dimensions, island species exhibited significantly longer GI length-to-SVL (F_7,136_ = 18.48, *p* < 0.001) and GI length-to-GI mass (F_7,136_ = 7.76, *p* < 0.001; Figure 3) ratios. Similar results were obtained when SVL was used as a covariate (ANCOVA for GI length: F_7,135_ = 62.91, *p* < 0.001).

### 3.2. Digestive Enzyme Activities

**Figure 4:**
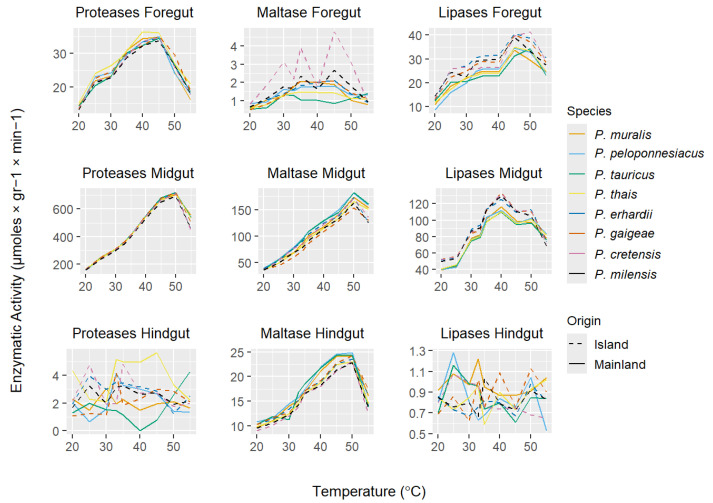
Curve plots showing the activity of proteases, lipases, and maltase (μmoles × gr^−1^ × min^−1^) within the three gut regions in eight *Podarcis* species. Solid lines denote mainland species (i.e., *P. muralis*, *peloponnesiacus*, *P. tauricus*, *P. thais*), while dashed lines denote islanders (i.e., *P. erhardii*, *P. gaigeae*, *P. cretensis* and *P. milensis*).

Digestive enzyme activities were highest in the midgut and lowest in the hindgut across all studied species and temperatures (all *p* < 0.001; Figure 4). In addition, enzyme activity increased significantly with temperature for all species up to 50 °C, followed by a significant decline at 55 °C (Figure 4). However, this decline was more evident on island species than mainland ones (midgut; proteases: 34% versus 24%, F_1,137_ = 383.80, *p* < 0.001; lipases: 31% versus 20%, F_1,137_ = 4.69, *p* < 0.001; and maltase: 21% versus 11%, F_1,137_ = 280.70, *p* < 0.001).

The comparison of peptidase and glucosidase activities in the foregut (proteases: F_1,7_ = 0.51, *p* = 0.834; maltase: F_1,7_ = 0.53, *p* = 0.811), midgut (proteases: F_1,7_ = 1.27, *p* = 0.260; maltase: F_1,7_ = 10.95, *p* < 0.001), and hindgut (proteases: F_1,7_ = 2.61, *p* = 0.011; maltase: F_1,7_ = 0.92, *p* = 0.493) revealed no or minimal interspecies differences across temperature treatments. The few significant differences detected, such as increased maltase activity in the midgut, appeared to occur randomly, without any environmental or phylogenetic signal (Figure 4). Similar results were observed when considering morphological variables such as body length and gastrointestinal tract size, with no significant relationships found (all *p* > 0.05).

**Figure 5:**
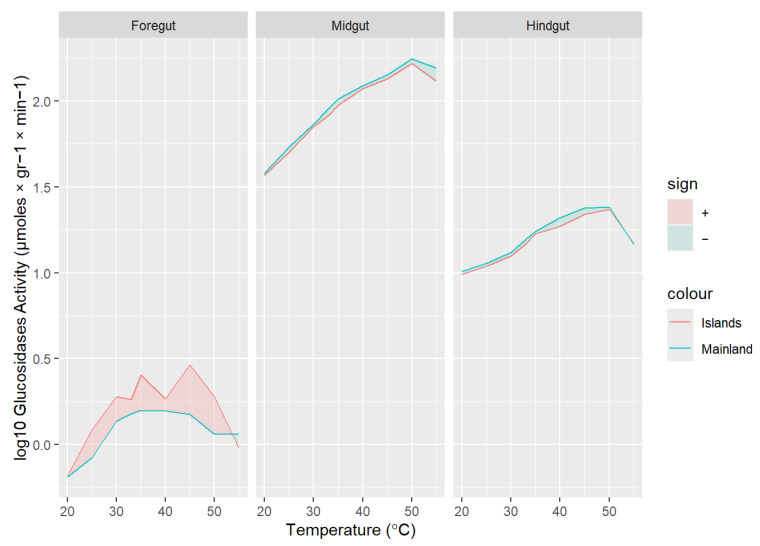
Density curve plots of lipase activity log10 transformed within the three regions of the gastrointestinal tract, illustrating the effects of increasing temperature on enzyme activity. The plot highlights differences between insular (*P. erhardii*, *P. gaigeae*, *P. milensis*, *P. cretensis*; red) and mainland (*P. peloponnesiacus*, *P. tauricus*, *P. thais*, *P. muralis*; blue) *Podarcis* species.

Regarding lipase activity, a distinct pattern emerged: island species tended to cluster together in both the foregut (F_7,136_ = 41.93, *p* < 0.001) and midgut (F_7,136_ = 12.24, *p* < 0.001), exhibiting increased activity across all tested temperatures (Figure 4 and Figure 5), whereas mainland *Podarcis* species formed a separate group. This grouping pattern remained even after controlling for snout-vent length (SVL), body mass, and gastrointestinal tract length (all comparisons *p* < 0.001). No significant differences in lipase activity were observed in the hindgut (F_7,136_ = 1.43, *p* = 0.191).

### 3.3. Phylogenetic Signal Effects

**Figure 6:**
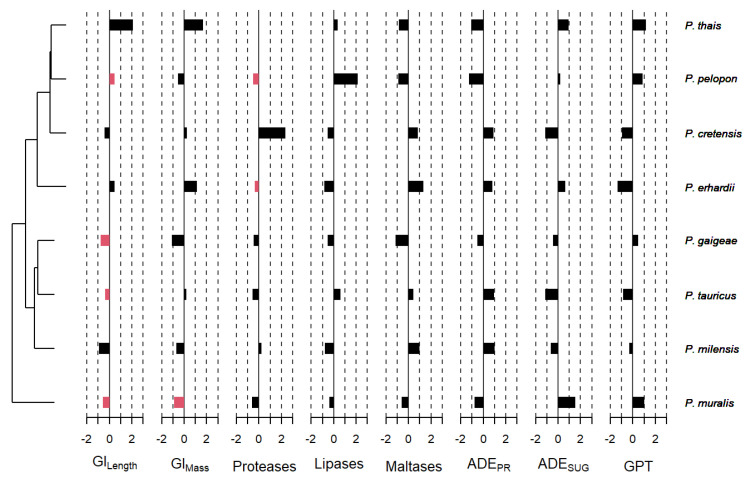
Phylogenetic tree reconstructed by maximum likelihood from the mitochondrial protein encoding cyt b and a partial sequence of the nonprotein coding mitochondrial 16S [30] for the eight *Podarcis* species. The bars give the score/signal of each taxon on the first principal component of the phylogenetic PCA. Red bars indicate a phylogenetic signal in the trait.

To account for phylogenetic effects on enzyme activities, we repeated all analyses, integrating physiological data with the phylogenetic tree. Our findings showed no statistical evidence of a phylogenetic signal for any tested trait (Table 1 and Figure 6). Accordingly, all phylogenetic correlograms were either flat and nonsignificant or exhibited weak positive autocorrelation.

## 4. Discussion

Ambient environmental temperature is a key factor affecting directly or indirectly species’ overall biology [51]. This is especially true for ectotherms, such as lizards, which rely on external heat sources to regulate their body temperature and optimize their physiology, metabolism and development [52]. Our in vitro laboratory experiments showed that increasing temperatures affect the activities of digestive enzymes (protease, lipase, and maltase) across all segments of the gastrointestinal tract. However, the magnitude of this response varied among species and was independent of phylogenetic relationships (i.e., closely related species within the same clade often displayed substantial differences, whereas adjacent clades were more likely to share similar trait values). As anticipated, following the study of Pafilis et al. [4], the island species, *P. cretensis*, *P. erhardii*, *P. milensis* and *P. gaigeae*, exhibited a more pronounced increase in lipase activity in relation to temperature, while no consistent grouping was observed for protease and maltase activities. Additionally, island lizards were found to have relatively longer and heavier gastrointestinal tracts in proportion to their body size, supporting our hypothesis and previous findings on the digestive systems of lacertid lizards inhabiting island environments [12,53].

Arthropods, particularly insects, constitute the major component of the *Podarcis* lizards’ diet [33,54,55,56,57], although both inter- and intraspecific dietary variations have been reported, likely due to geographical and/or seasonal differences in prey composition and availability e.g., [33,55]. These shifts in prey consumption allow lizards to meet their energy requirements under resource-limited conditions. In line with the typical enzymatic profile of carnivorous species, we found that *Podarcis* lizards exhibit high protease and lipase activity, but low maltase activity across all segments of the gastrointestinal tract. Members of the genus *Podarcis* are predominantly insectivorous, with insects providing a rich source of protein and fatty acids, but being relatively low in digestible fiber [58,59,60]; the primary structural carbohydrate in insects is insoluble chitin, which is concentrated in their exoskeleton [60,61]. The observed enzymatic profile therefore suggests an adaptation facilitating the efficient digestion of protein and lipid-rich diets. Notably, digestive enzyme activity was higher in the midgut, where most enzymatic breakdown occurs, and in the foregut, where initial hydrolysis begins [62,63]. In contrast, enzyme activity was lower in the hindgut, which in carnivorous/insectivorous reptiles is primarily involved in nutrient and water absorption, rather than enzymatic digestion [64,65].

As a temperature-dependent process, the efficiency of protein, lipid and sugar digestion is highly sensitive to changes in body temperature, primarily due to its influence on food transit time, enzymatic breakdown, emulsification, and nutrient absorption [4,39,66,67,68]. While a shortened transit time can reduce digestive efficiency by limiting the duration of food processing, increased enzyme activity and other compensatory digestive mechanisms at higher temperatures may help maintain overall digestive performance. Classical enzymatic theory predicts that enzyme activity generally increases with temperature up to an optimal threshold, beyond which activity plateaus or declines [69,70] as enzymes begin to denature and lose functionality [38,71]. Our in vitro study utilized a temperature gradient ranging from 20 °C to 55 °C, reflecting the range of temperatures that lizards are likely to experience in their natural environments. Consistent with previous studies showing that digestive enzymes become heat-sensitive at temperatures above 45 °C [38,39], we observed no loss of activity for proteases, lipase, and maltase up to 40 or even 45 °C. This suggests a high degree of thermal plasticity in digestive enzyme function.

The comparative analysis of protease and maltase activity within the three regions of the digestive tract revealed no consistent clustering of enzymatic activity among species, with only random differences detected (Figure 4), indicating a general similarity between mainland and island populations and no evidence of species- or population-specific adaptations to local environmental conditions. These findings align with previous studies on enzymatic activity in ectotherms that support the hard-wired hypothesis [72,73]. Examining the apparent digestive efficiency in a subset of these species across a range of temperatures, Pafilis et al. [4] identified two distinct groups, with island taxa exhibiting approximately 30% higher efficiency for proteins (ADE_PROTEINS_) and 10–20% shorter gut passage time (GPT), although no differences were observed in ADE_SUGARS_. Given the typically negative correlation between GPT and digestive efficiency, these findings may appear contradictory. Potential explanations for the increased ADE_PROTEINS_ are either enhanced digestive enzyme activity or improved nutrient absorption in the gut. On one hand, our data allow us to reject the first hypothesis, as no such pattern of differences in protease activity was detected among mainland and island species across all temperature regimes. On the other hand, gut morphology differed significantly between island and mainland taxa. Island taxa exhibited gastrointestinal tracts that were, on average, 10% longer and 18% heavier—a proxy of increased gut surface area or thicker intestinal mucosa and muscle layers [74,75]—relative to body size, compared to their mainland counterparts (Figure 3). These morphological differences provide strong evidence that increased nutrient absorption, rather than differences in enzymatic activity, might underlie the higher digestive efficiency [62,76] of proteins and sugars observed in insular *Podarcis* lizards [4]. Notably, protease and maltase activities in mainland lizards showed a weaker temperature dependence at the upper end of the tested range, maintaining relatively higher activity at high incubation temperatures compared to island species (Figure 4). This pattern suggests convergent adaptation among mainland species to more variable thermal environments, which may select for broader thermal tolerance in digestive enzymes. Interestingly, this enhanced thermal resilience in enzyme activity was observed even though mainland *Podarcis* species are phylogenetically nested within clades [30] containing island congeners, indicating that these adaptations are likely a response to environmental conditions rather than phylogenetic constraints.

Contrary to the patterns observed for proteases and maltase, lipase activity showed an increase of approximately 13% across all temperatures in island taxa compared to mainland *Podarcis*. Lipid digestion begins in the stomach through the action of gastric lipase which breaks dietary lipids into smaller droplets, and continues in the duodenum [77], where emulsification and micelle formation facilitate passive absorption at the intestinal brush border [78,79]. Given the longer gastrointestinal tracts in island lizards and the high metabolic cost of lipase production [80], this upregulation is intriguing, as strong selection for elevated lipase activity might not be expected under such conditions. However, island populations are known to often experience lower and more unpredictable food availability than their mainland kins [4,81]. In this context, efficient lipid digestion may play a disproportionately important role in energy acquisition to support energy-consuming processes such as growth and reproduction. Fats are the most energy-dense macronutrients, yielding over twice the energy per gram compared to proteins or carbohydrates. Under such resource-scarce and fluctuating conditions, maximizing the extraction of fatty acids from available prey could confer a significant selective advantage; a digestive strategy reported in several taxa [4,12,82,83,84].

Alternatively, enzyme activity, including that of lipases, is often tightly correlated with dietary substrate availability [80,85,86]. Thus, the elevated lipase activity observed could reflect dietary differences among the eight studied sites and species, potentially due to variations in lipid content across arthropod prey [87]. The upregulation of lipases, in conjunction with longer and heavier gastrointestinal tracts in island populations (Figure 3), may, therefore, represent a compensatory strategy to enhance fat utilization. However, because we lack direct empirical data on dietary composition for the studied populations, such interpretations remain speculative, and should be tested explicitly in future studies combining enzymatic assays with direct dietary analyses. Notably, previous work has shown that island species exhibit higher digestive efficiency for proteins, likely as a result of slower gut passage times [4]. In contrast, lipid digestion may rely more strongly on enzymatic activity, particularly when micelle formation is limited due to suboptimal bile salt availability or low dietary emulsifiers in insular diets [88,89]. Thus, the increased lipase activity may not simply reflect higher lipid intake, but rather a physiological need to overcome digestive constraints specific to lipid processing in resource-poor environments.

## 5. Conclusions

While several studies have shown that reptiles, including lizards, are generally very efficient in extracting energy from their food, our understanding of how reptiles meet their nutritional needs (proteins, lipids and sugars) and how temperature may influence this balance is still rather limited, in most contexts [90]. Here, we examined the thermal sensitivity of digestive enzyme activity across several closely related *Podarcis* species, integrating physiological and morphological data within a phylogenetic framework. We found that enzyme activity generally increased with temperature, but island species displayed steeper declines in activity beyond 50 °C (30% vs. 19% decrease), suggesting reduced thermal tolerance compared to their mainland counterparts. These differences, together with distinct gut morphologies, gut passage time and digestive efficiencies [4], point toward divergent adaptive strategies [76] shaped by the contrasting environmental pressures of mainland and island habitats, especially in terms of food availability and thermal variability. Given variations in global temperatures, our findings highlight the fact that even closely related ectothermic species can exhibit markedly different physiological responses to rising temperatures and different adaptive capacity. Should ambient temperatures rise and heatwaves become more frequent and intense, the digestive efficiency and nutrient acquisition strategies of reptiles may be increasingly challenged, particularly in insular populations with narrower thermal tolerances and less genetic diversity upon which selection may act. Understanding these physiological constraints and adaptive responses is crucial for predicting species resilience and distribution under future climate scenarios [91] and underscores the importance of integrating digestive physiology into broader discussions of reptile vulnerability and climate adaptation.

## Figures and Tables

**Table 1:** Measurements and tests of the phylogenetic signal for 10 physiological and morphological traits with two methods (Pagel’s λ and Abouheif’s *C*_mean_). The values of ADE and GPT were retrieved from the study of Pafilis et al. (2007) [4]. Bold values indicate a statistically significant phylogenetic signal.

Trait	*C*_mean_	*p*-Value	λ	*p*-Value
Body Length (SVL)	**0.309**	**0.019**	0.001	0.999
Body Mass	−0.056	0.279	0.001	0.999
GI Tract Length	**0.193**	**0.027**	0.001	0.999
GI Tract Mass	−0.115	0.347	0.001	0.999
Protease Activity	−0.042	0.234	0.001	0.999
Lipase Activity	−0.167	0.489	0.001	0.999
Maltase Activity	−0.157	0.557	0.001	0.999
ADE_PR_	−0.069	0.311	0.001	0.999
ADE_SUG_	−0.051	0.263	0.452	0.999
Gut Passage Time	−0.133	0.414	0.001	0.999

## Data Availability

The data presented in this study are available on request from the corresponding author.
